# Reported roles of care partners in a specialized weaning centre—perspectives of patients, care partners, and health care providers

**DOI:** 10.3389/frhs.2024.1439410

**Published:** 2024-10-30

**Authors:** L. Istanboulian, A. Gilding, L. Hamilton, T. Master, S. Bingler, M. Hill, S. Isani, S. Kazi, S. Coppinger, K. Smith

**Affiliations:** ^1^ Daphne Cockwell School of Nursing, Faculty of Community Services, Toronto Metropolitan University, Toronto, ON, Canada; ^2^ Michael Garron Hospital, Toronto East Health Network, Toronto, ON, Canada; ^3^ Institute of Health Policy, Management, & Evaluation, Dalla Lana School of Public Health, University of Toronto, Toronto, ON, Canada

**Keywords:** patient engagement, care partners, persistent critical illness, safety, critical & intensive care

## Abstract

**Background:**

Care partners are individuals chosen by a person with an illness to support their care during hospitalization. Patients with persistent critical illness have longer than average critical care admission and often other conditions including dysphagia, communication vulnerability, severe physical deconditioning, the need for an artificial airway, and difficulty weaning from invasive mechanical ventilation. Family presence has been identified as important for patients experiencing persistent critical illness in specialized weaning centers. Despite this, the role of care partners in clinical settings for patients with persistent critical illness has not been fully characterized, particularly from the perspectives of patients, care partners, and health care providers. The aim of this study was to gain insights into the roles of care partners during persistent critical illness from the perspectives of patients, care partners, and health care providers.

**Methods:**

We used qualitative descriptive methodology including semi-structured interviews and content analysis. Interviews were audio recorded and transcribed verbatim. Included participants (*n* = 30) were patient survivors (*n* = 7), care partners of patient survivors (*n* = 9), and professionally diverse health care providers (*n* = 14) of adult patients with persistent critical illness from two specialized units in one community academic hospital in Toronto, Canada.

**Results:**

Participants across all groups described care partner roles that included physical, mental health, cognitive, social, and spiritual support of the patient, including the perceived role of safeguarding the multiple dimensions of care for the patient who is experiencing persistent critical care in specialized care settings.

**Discussion:**

The results of this study are being used to co-design, implement, and evaluate a sustainable care partner program that is acceptable, appropriate, and feasible to implement in clinical settings where the care of patients with persistent critical illness occurs.

**Reporting method:**

Consolidated criteria for reporting qualitative studies (COREQ).

## 1 Introduction

A care partner is an individual (often a family member) chosen by a person with an illness to support their care (1). The roles care partners take on depend on the person's needs but can include helping the care team to better understand the person's needs and preferences, monitoring disease-specific signs and symptoms, supporting physical care including feeding, bathing, and mobility, and organizing follow up appointments and care in the community (1). Emerging policies and research for the integration of care partners in complex and acute care centres recognize the unique positioning of care partners (who are often family members) as sources of insight and resilience in supporting the care quality and safety (2, 3). Particularly as COVID-19 pandemic conditions changed family presence policies and visitor restrictions, the importance of defining care partners, with a role beyond that of a visitor, emerged (2, 4, 5). Recommended foundational preferences from Healthcare Excellence Canada for the inclusion of care partners in facilities include clearly differentiating them from visitors, recognizing their value, and ensuring they play a role in the development of policies pertaining to care partner programs (2). The COVID-19 pandemic and research emerging during, and since, has illuminated the critical work of family members in protecting the safety of vulnerable patients who are more likely to experience safety gaps in their care (6).

An understudied patient population who experience significant health vulnerabilities and risks to safety both during and post-hospitalization are those with longer than usual intensive care unit (ICU) admissions, otherwise known as persistent critical illness. Up to 10% of patients in ICU experience persistent critical illness (7). Patients with persistent critical illness experience organ dysfunction, prolonged dependence on mechanical ventilation, and the need for tracheostomy (7). In regions where they are available, patients with persistent critical illness are often admitted to specialized weaning centres (8–10). Family presence has consistently been identified as important for patients experiencing persistent critical illness in specialized weaning center, however the specific ways in which they support patient care and safety is not yet clear (10–12). Furthermore, a scoping review of patient and family involvement in ICU identified research gaps including limited understanding of the bidirectional implications between patient and family involvement and the interprofessional team (13).

Although we know patients, family, and health care providers report the importance of family presence for patients experiencing persistent critical illness, little is known about the perception of the care partner role from the perspectives of patients, care partners, and health care providers. A fulsome understanding of the roles that care partners take on during persistent critical illness can therefore help improve how teams and policies can support their work, and ensure policies include their unique perspectives, and improve patient care quality and safety. This research will contribute to a body of knowledge that will aid care partner program design, implementation, evaluation, and potential spread. Thus, the primary aim of this study was to gain insights from the lived experiences of patients, care partners, and health care providers about the roles of care partners during persistent critical illness. The secondary aim was to compare care partner role descriptions between participant stakeholders.

## 2 Methods

### 2.1 Design

We conducted a qualitative descriptive study including the use of semi-structured interviews to understand care partner roles through participants’ descriptions and use this knowledge to improve programs (14, 15).

### 2.2 Study setting

The study was conducted at a large, 500-bed, urban Community Academic Hospital located in Toronto, Canada. This setting has two units where patients with persistent critical illness are managed, including an eight-bed adult specialized weaning program that is set within an acute inpatient respiratory ward and a six-bed adult long-stay ICU program that is set within a medical surgical ICU. Both units have diverse multiprofessional care team members, including intensivists (i.e., medical physicians with critical care speciality), respirologists, nurse practitioners, and nursing and allied care. Both programs also admit ICU patients from external hospitals across Ontario.

### 2.3 Participants & sampling

We used a multi-modal and convenience recruitment strategy including the use of study posters, announcements at unit huddles, and members from patient and care partner circles of care identifying potential participants. Of all the potential patient and care partner participants approached, only two did not participate due to medical instability. Recruitment continued until we perceived our recruitment targets were met in terms of variation in participant characteristics, meaning that we had recruited sufficiently from the diverse professional body in both units, and as many patient and care partners as we were able to during the study period. We stopped recruitment when we felt we had understood the roles of care partners and had achieved sufficient information power for the relatively narrowly focused study aim and the specificity of our study participants (16).

### 2.4 Inclusion/exclusion criteria

Inclusion criteria for patient participants comprised (1) 18 years or older; (2) currently admitted to or recently discharged from the study setting (within 1 year, or longer if able to recall experiences); (3) medically stable according to medical provider; (4) able to communicate by some means (e.g., gestures, augmentative or alternative communication, phonation); (5) some English speaking at minimum; (6) able to provide informed consent (i.e., able to understand and appreciate the consequences of their decision to participate or not participate in the study).

Inclusion criteria for care partners comprised (1) 18 years or older; (2) a formal care partner (identified as a person who has a designated role for the patient, beyond that of a visitor) of a patient who is currently admitted to or was recently discharged from the study setting (within 1 year or longer if able to recall experiences); (3) able to provide informed consent (i.e., able to understand and appreciate the consequences of their decision to participate or not participate in the study), and able to speak some English. All care partner participants played active roles in the care of patients in the programs. care partners were only approached for recruitment if their related patient agreed.

Inclusion criteria for health care providers comprised only that they were employed at the study setting in the past year and had some experience working with care partners. There was no exclusion criteria for health care providers.

### 2.5 Data collection

Following informed consent, some members of the research team (LI/SB/TM/AG) conducted interviews using a semi-structured interview guide (See Supplementary Material S1) developed iteratively by the full research team. During the first two interviews we reviewed findings which confirmed no changes to the interview guide were required. Demographic information was collected at the time of the interview. Interviews were conducted in person with 17 (57%) participants, over the phone with 7 (23%) participants, or by using a videoconferencing platform (i.e., Zoom) with 6 (20%) participants according to their preference and availability. Interviews were between 20 and 62 min long (average 40 min), digitally recorded, and transcribed verbatim and coded using NVIVO (Version 14, Lumivero, Denver, CO). No repeat interviews were conducted. All patient and care partner participants were offered to have interviews conducted individually or in dyads, and therefore, 12 (75%) individual interviews were done, and 2 (4 participants, 25%) interviews were conducted as dyads per the preference of the participants. Preliminary discussions were used to establish a relationship with each participant and comfort with the subject area. Some participants were previously known to the interviewers. Notes were made on participant reactions to questions, responses, meaningful pauses, and reflections not otherwise captured by the digital recording or transcription.

### 2.6 Data analysis and reflexivity

Data analysis employed a team-based directed inductive content approach (17). Inductive content analysis included four distinct stages: (1) Decontextualization (meaning units identified (2) Recontextualization (including the content, whereby the meaning units were compared with the original data), (3). Categorization (where we identified convergences and divergences in the subcategorization and subjects), and 4. Complication (where we drew conclusions from the overall categories and sub-categories, including comparing across groups) (17). Throughout the process we worked in pairs to iteratively create and define categories. The team met regularly to compare findings, discuss and revise definitions, and to develop sub-categories within the main codes that comprehensively described the participant reported care partner roles.

The research team has extensive experience working with patients and family experiencing persistent critical illness and engaged in reflexive discussions during the data collection period and analysis. LI, SB, TM, and AG all work at the study setting and conducted the interviews. LI is a Nurse Practitioner, SB is a nurse and works in the Patient Relations Department, TM is a clinical educator, and AG works at the study setting as a research student. Three interviewers identify as women, and one as a man, and all have experience with semi-structured interview methods. Through reflexive group discussion we were able to discuss the interview transcripts, quotes, and contextual nuances of the roles described. These reflexive sessions between and at the conclusion of the interviews helped the team consolidate the main and sub-categories and compare results across the various participant groups.

### 2.7 Ethical considerations

Ethics approval was obtained from Michael Garron Hospital (883-2211-Mis-391) and Toronto Metropolitan University (REB 2023-357). Informed written consent was obtained ahead of the interview with assent audio-recorded before the interview commenced. Participants were coded by a number on the transcripts [e.g., HCP 001, Patient 002, or CP (for care partner) 003, etc.] to preserve anonymity.

### 2.8 Rigor

In accordance with recommendations for transparent and comprehensive reporting, we used the Consolidated Criteria for Reporting Qualitative Research guidelines to describe our methods and findings (18). To enhance the credibility and trustworthiness of the analysis a code book (See Supplementary Material S2 for final code definitions) and audit trail were created (19, 20).

## 3 Results

### 3.1 Participant characteristics

We recruited 30 participants (Table 1). Of these, 7 (23%) were patients, 9 (30%) were care partners, and 14 (47%) were health care providers. Participants were diverse in terms of self-identified gender, relationship to the patient, and professional role in the setting.

**Table 1:** Participant demographics (*N* = 30).

Participant	Demographics	Category	n (%)
Patient			7 (23)
	Gender	Woman	3 (42)
		Man	4 (58)
	Age	Years (mean, sd)	61.3 (14.7)
	Race	Caucasian	6 (86)
		Asian	1 (14)
Care Partner			9 (30)
	Gender	Woman	7 (78)
		Man	2 (22)
	Age	Years (mean, sd)	59 (13.7)
	Relationship	Partner/spouse	5 (56)
		Child	2 (22)
		Parent	2 (22)
	Race	Caucasian	7 (78)
		Asian	2 (22)
Health care provider			14 (47)
	Gender	Woman	10 (71)
		Man	4 (29)
	Age	Years (mean, sd)	44.9 (11.4)
	Race	Caucasian	9 (64)
		Asian	3 (21)
		Black	2 (14)
	Profession	Nurse	5 (36)
		Speech Language	2 (14)
		Physician	2 (14)
		Social Worker	2 (7)
		Physiotherapist	1 (7)
		Occupational Therapist	1 (7)
		Respiratory Therapist	1 (7)

### 3.2 Reported roles

Patients, care partners, and health care providers reported a wide range of activities that care partners performed in support of the patient experiencing persistent critical illness (Table 2 and Supplementary Material S3 for additional quotes). Although there were some commonalities in participants’ descriptions of the actions or roles of care partners, there were also some roles uniquely reported by some participant groups but not by others. Reflexive discussion of the coding categories led to our research team categorizing the support actions according to the type (i.e., physical, mental health, social, cognitive, and spiritual) of support provided, as this was the predominant way participants reported care partner roles. Health care providers additionally expressed that care partner roles vary from person to person and change over time with increasing comfort. The findings below are organized by the type of support provided to highlight convergences and divergences in care partner support roles during persistent critical illness.

**Table 2:** Reported roles of care partners as reported by patients, care partners, and health care provider participants.

	Patients	Care partners	Health care provider
Physical	Personal care and physical therapy	Personal care and physical therapy	Personal care and physical therapy
	Safety and surveillance	Safety and surveillance	Safety and surveillance
	Access to outside	Access to outside	Access to outside
	Access to equipment and food from home		
			Care planning and continuity of care
Mental health	Encouragement	Encouragement	Encouragement
	Emotional support	Emotional support	Emotional support
Social	Companionship	Companionship	Companionship
	Gatekeeping	Gatekeeping	Gatekeeping
	Sense of community	Sense of community	
Cognitive	Health literacy, decision making, advocacy	Health literacy, decision making, advocacy	Health literacy, decision making, advocacy
	Communication support	Communication support	Communication support
			Information transfer
			Delirium management and prevention
Spiritual	Normalcy	Normalcy	Normalcy
	Support values	Support values	Support values
	Manage outside life		

#### 3.2.1 Physical roles

Physical roles were defined as roles and actions care partners took to support the physical care, and physical safety of the patient. Physical roles were also ways the care partners enhanced physical access to the items or experiences that were needed or valued by the patient. Participants in this study described care partner physical roles in relation to 1. Personal care physical therapy, 2. Safety and surveillance, 3. Access to personal items and outside, and 4. Care planning and continuity of care.

##### 3.2.1.1 Personal care and physical therapy

Patients, care partners, and health care provider participants all described physical care roles for care partners. These roles included hair washing, body-care, assisting with activities of daily living, and physical therapy. Patients and care partners also reported physical care activities that included touch such as massage. Participants from all three groups reported that care partner physical care “supplemented the care” provided by nurses and other members of the health care team (HCP 005) and was important because health care providers do not always “have the manpower” (HCP 009) to manage all patient care needs in a timely manner. Both patient and care partner participants noted this facilitating role.

“Let’s be honest, I guess if it’s busy, are we going to wait out an hour for a nurse? [Be]cause my mom’s going to help me put my pants on.” (Patient 005)

“So, I said probably instead of waiting for the nurse to put the food, you know, the feed. Yeah, probably I can do that.” (CP 007)

The critical importance of providing physical care beyond what the health care team was able to provide was emphasized by one care partner, who stated:

“I think had we not been there pushing him to get into his chair, communicating with him, I think he would be in worse shape than he is.” (CP 006)

##### 3.2.1.2 Safety and surveillance

Patients, care partners, and health care providers emphatically described the role care partners played in supporting the safety of patients through surveillance of the patient and their surroundings. From the patient's perspective, this included double-checking medications, scheduling tests, and ensuring continuity of care.

“She was so diligent about checking the meds, checking with doctors and nurses and making sure that the delivery of health care was exceptional from the hospital.” (Patient 001)

Similarly, one care partner reported:

“The only thing that I said yesterday is that many, many times I saw the call bell unplugged and asked why is that?” (CP 007)

This quote emphases the safety surveillance role that care partners play in assessing the physical environment. Care partners detect and report fractures in care and protocols (e.g., having call bells plugged in and at the bedside) that are meant to keep patients safe.

Several health care providers described the roles care partner played in maintaining the safety of patients in these settings. One health care provider summarized many aspects of this role, stating,

“And there’s more than that, as well. So, I’ve also seen them, for example, draw attention to issues to the medical care team about changes in a patient’s condition, about things from changes in respiratory rate, agitation, to a skin ulcer, for example, or things like that. So, they’re also part of increasing the vigilance in the care of the patient, which I think is also critical.” (HCP 012)

The vulnerability and medical fragility of these patients as they recover from critical illness demands an approach to care that includes care partners, particularly if they can detect and report issues as is described by the quote above.

##### 3.2.1.3 Access to personal items and outside

Patients, care partners, and health care providers all reported that care partners often improved patient access to spaces outside their rooms, a significant role, given prolonged admissions in these settings for patients with persistent critical illness. One patient participant who had been in hospital for over a year stated:

“They [care partner] would take me outside. I love the outdoors … I could smell the flowers and feel the fresh air. It was so nice.” (Patient 008)

Only patient participants, however, reported that care partners provided access to personal equipment needed for therapy and care such as “footwear” (Patient 008) from home and even equipment from hospital clean utility rooms such as “urinals.” (Patient 005). Patients also reported that once they were able to eat by mouth, care partners would bring in preferred food from home.

##### 3.2.1.4 Care planning and continuity of care

Health care providers uniquely reported that care partners also played an integral role in care planning during the patients’ admission, providing continuity of care, and transition support.

“Because they’re thinking ahead of us when we go home—‘What does home look like—if we need to do this’. So, some will ask, ‘Can you teach me how to do this?’ And we’ll walk them through certain things.” (HCP 010)

And,

“They’re always included the care. So, it’s nice to have the continuity of care, you know, what they were doing there.” (HCP 006)

These statements highlight the value health care providers placed on the role of care partners in the planning and provision of safe care both during admission and during preparations for discharge planning and transition home.

#### 3.2.2 Mental health roles

Mental health roles were defined as roles and actions care partners took to support the mental health wellness and experience of the patient. These roles included proactive (e.g., encouragement) and reactive (e.g., emotional support) actions towards the patient intended to support their emotional well-being. Participants in this study described care partner mental health roles in relation to providing 1. Encouragement, and 2. Emotional support.

##### 3.2.2.1 Encouragement

Patients, care partners, and health care providers ubiquitously reported the supportive role that care partners played in providing patients with encouragement and motivation. One patient participant called his care partner his “greatest champion” (Patient 007), and another who stated,

“Sure, it helped me. I can’t explain to you, but when she was beside me, I felt better. Encouraging me, saying you’re ok, you’re not sick. You’re coming home. [She] made me feel hopeful.” (Patient 004)

With the prolonged and uncertain course of persistent critical illness, care partners also provided encouragement and hope for recovery. For example, one care partner participant said:

“I think that emotionally, I think I helped her just to understand sometimes what was going on and just to constantly help her to stay hopeful.” (CP 009)

Health care providers similarly reported:

“They’re the encouragement and the cheerleaders in order to keep them going through everything.” (HCP 003)

##### 3.2.2.2 Emotional support

Patients, care partners, and health care providers also described multiple ways in which care partners provided emotional support to patients with persistent critical illness. Distinct from visitors, who were also described as important to patients, care partners were the people with whom patients could share their concerns and fears with.

“My [care partner] was a real source of emotional support and strength in that with … [them] I could vent or discuss things that I needed to get off my chest that you might not feel comfortable with a friend or visitor.” (Patient 005)

Care partners described the importance of supporting patients by protecting their “emotional well-being” (CP 011) and reducing anxiety.

“And look, my daughter probably didn't want to know as much as I did, but I would use what I knew to reassure her, give her hope, help her not to be anxious.” (CP 009)

Like care partners, health care providers emphasized the importance of care partners in reducing patient anxiety, particularly in advancing ventilation weaning trial times or time sitting up in a chair.

“The family members that are there help with their, with their trials, or reducing anxiety or fear or whatever.” (HCP 009)

Other health care providers described a more ambiguous but no less important role care partners play emotionally supporting patients with persistent critical illness,

“So, they are critical in ensuring that our patients’ mental health is well supported. And that’s a role that unfortunately, nobody else in the hospital can do. There’s only so many antidepressants you can give somebody. So, I think they’re critical in all of those aspects.” (HCP 012)

This statement emphasizes the potentially complementary and uniquely suited role of care partners to pharmaceutical agents in managing mental health needs of patients with persistent critical illness.

#### 3.2.3 Social role

Social roles were defined as roles and actions care partners took to support and manage the social needs and situation of the patient. Participants in this study described social roles of care partners in relation to 1. Providing companionship, 2. Creating a sense of community, and 3. Acting as gatekeepers.

##### 3.2.3.1 Providing companionship

Patients and family both reported that a key role of care partners was to provide company and companionship during admission, particularly important for these patients with longer than normal lengths of stay. One patient participant aptly described this, stating,

“It’s nice when you have somebody to talk to and it helps to have someone to talk to because you stay so long in the hospital. It was like our home. Same thing, you know what I mean.” (Patient 003)

Health care providers empathically described the role of care partners in protecting patients with prolonged admissions from social isolation.

“I think it’s very important to have people [at the] bedside—family members or friends. Because it can get really lonely in the hospital when you’re alone.” (HCP 009)

##### 3.2.3.2 Creating a sense of community

Patient and family participants were particularly attuned to the impact that the regular presence of care partners in the care setting had on creating a sense of community for patients with persistent critical illness.

“Yeah, but she knew about them [the staff] because I would talk about them. I could say so and so came and did physio with me or so and so came and gave me a bed bath and she knew who they were.” (Patient 008)

Her care partner also noted the impact of her regular presence on the unit, and knowledge of the staff, stating,

“And I think the fact that. I knew team members as well. I think I could speak to people because I came to know them, and they came to know me. And I think as I felt more comfortable, it helped her to feel more comfortable. She started to feel at home.” (CP 009)

The displacement of patients from their homes due to prolonged admission put them at risk for social isolation, mitigated in part by care partner companionship and the sense of belonging and community created through social connections shared between patients, care partner, and health care providers.

##### 3.2.3.3 Acting as gatekeepers

Patients, care partners, and health care providers all reported the role care partners played in monitoring and regulating would-be and actual visitors. In many cases, care partners would be the ones who took on the responsibility of restricting visitors if they were unwell or if the patient was fatigued. On this, one patient reported:

“She does all my dirty work telling my friends to stay away when I don't have the heart to do it.” (Patient 013)

And both care partners and health care providers similarly stated:

“But the same goes with company and visitors. If someone wants to come, everybody has been very good and they will always reach out and say, can we come over? It’s up to you if you don't feel like it. No one’s feelings are going to be hurt. You need to be proactive. So that’s where I feel like I'm stepping up and like, okay, I'll be the bulldog. You know.” (CP 012)

And,

“They are also the gatekeeper for visitors, they know who comes to visit and when they know they control those people who come to visit them.” (HCP 005)

#### 3.2.4 Cognitive roles

Cognitive roles were defined as roles and actions care partners took to support cognitive needs of the patient including understanding and directing care, as well as receptive and expressive components of communication. Participants in this study described care partner cognitive roles in relation to providing 1. Health literacy, decision making, advocacy, 2, Communication support, 3. Health information management, and 4. Delirium management and prevention.

##### 3.2.4.1 Health literacy, decision making, advocacy

Participants from all stakeholder groups reported that a key role of care partners in this clinical setting was to support patient decision making. During acute critical illness this role more commonly falls to the substitute decision maker or power of attorney if one is appointed. During persistent critical illness, patient level of consciousness is improved, and at worse fluctuates. This makes the role of supporting decision making more complicated, but still an important one to consider when considering the role of care partner. One patient, who was unable to vocalize at the time of the interview, wrote:

“[writes] [My care partner] talks to the doctors and nurse practitioner to get updates while I'm half-awake most of the time. He is my voice and my medical advocate.” (Patient 010)

Similarly, a care partner explained:

“And sometimes I would just explain a little bit of what they mean. The physicians were pretty good at explaining and [the nurse practitioner was] always good at sort of interpreting what they said for us. I also did that for her sometimes, and I think that also helped her. She’s also actually kind of a shy person and not one to always speak up for herself.” (Family 009)

Health care providers described another dimension of decision-making support and advocacy from the care partner resulting from their knowledge of the patient and the continuity of their care role over a prolonged period and settings. One health care provider described this role like this,

“They will, you know, advocate like say, the patient, you know, doesn’t want to take a certain medication at this time, maybe, because it makes them you know, like, the side effects aren’t too good for them. So, they will ask us if we can, you know, like, hold it, or reschedule it, or talk to the doctor about changing it.” (HCP 001)

##### 3.2.4.2 Communication support

Related to decision making and advocacy, most participants across all groups reported the role care partners play in supporting patient communication. Patients focussed heavily on the expressive components of communication. For example, one participant emphasized the vulnerability felt by patients with communication disability during persistent critical illness.

“And I think also when you're a patient you can’t especially because while I had trouble speaking and so you can't really advocate for yourself very well. And even if you can speak, every patient is different, but you're in a vulnerable situation.” (Patient 008)

Care partners similarly described the role they played interpreting the communication attempts made by patients and ’speaking for’ their loved ones.

“And I also found that when my husband couldn’t communicate either because he was on a ventilator, he had been trached before he came here. But even with the trach, he couldn't, you know, talk, but you could at least see his lips move. You know, you're really going to need that care partner to help interpret and speak for the patient, because they, in many cases, will not be able to do that for themselves.” (CP 002)

Health care providers shared this view as well, reinforcing the importance of the role care partners play in communication support for patients experiencing persistent critical illness. One health care provider stated,

“And they are also the voice of the patient. And then often patients are not able to speak for themselves, or able to navigate the system because of everything that they’re dealing with from a health-related standpoint.” (HCP 012)

##### 3.2.4.3 Information management

Health care providers described two additional dimensions of cognitive support of care partners including information management. Again, due to the protracted illness and length of stay, and impact on patient cognition and memory, care partners had information related to medical history that were not in the patient chart but are important to safety.

“Then again, they might know certain things about the patient’s personality, care needs. Physical medical history, things that would, you know, play a role in the patient’s health and safety.” (HCP 002)

##### 3.2.4.4 Delirium management and prevention

Health care providers also uniquely described the role care partners play in “orienting” (HCP 005) patients experiencing persistent critical illness, and the mitigating effect of their presence and activities on delirium. For example, one participant described how care partners can supplement the care team's role in orienting patients to a daily calendar or other “cognitive stimulating activities” (HCP 014).

#### 3.2.5 Spiritual roles

Spiritual roles were defined as roles and actions care partners took to support and manage the spiritual care of the patient, including their quality of life and definition of self in relation to others. Participants in this study described spiritual roles of care partners in relation to supporting (1) Normalcy, (2) Supporting values, and (3) Managing outside life.

##### 3.2.5.1 Normalcy

All groups reported the care partners role in supporting a sense of normalcy for patients experiencing persistent critical illness. Prolonged institutionalization and shifts in identity for both the patient themselves and in relation to others experienced during persistent critical illness underscores this role as uniquely critical for care partners to fulfil. Patients, for example, described how important it was for them to hear about life outside of their current medical concerns.

“It’s huge … with everything that happened and everything that we're enduring there’s enough heaviness going on, so it’s nice to keep things light. It’s nice. It’s nice not to have the conversation be 90% so what did the doctor say, what did the nurse say, what did the physio say, are what did they say about next steps … Let’s have some what’s going on at home.” (Patient 005)

Care partners in turn described how they preserved bonding activities with patients. For example, one care partner participant reported,

“We've become very close. I love her very much. And, uh, I just try to be around and do little things every day, like foot rubs and hairdos and yeah, watching our favorite music videos and stuff like that.” (Family 014)

Health care providers described the ways in which care partners would alter the physical environment to reflect the individual patient, integrating personal elements such as family photos and cards.

##### 3.2.5.2 Supporting values

In the same way that supporting a sense of normalcy was achieved by conversation and activities with care partners, patients described that these acts also reflected and supported their values, which they found protective and encouraging. To illustrate this, one patient reported,

“I suffered vision loss. And I couldn't really even hold a book, so they would read to me. That was so helpful. She brought me some drawings that people, my nieces, and nephews had done, and she would tape them to the wall. And I could look at them and be encouraged just by looking at them.” (Patient 008)

The importance of sharing with the care team who the person is, and what they value was reported by care partners, implying a state of departure from what the person was ‘like’ and how they are as they slowly recover from persistent critical illness. One family member described this role like this,

“And so, you know, sort of trying to fill in some of this stuff about it to give you a more of a 360 view that when I say you, I'm telling the medical team that you do more of a 360 view of who this person is in the bed.” (CP 002)

Beyond sharing values and protecting a sense of normalcy for the patient and relations, health care providers identified that prayer with the patient was for some an important role care partners played with patients experiencing persistent critical illness, especially for “patients that have very strong faith.” (HCP 007)

##### 3.2.5.3 Managing outside life

Only patient participants described the role care partners played in managing their outside life. These included maintaining domestic and financial responsibilities that patients themselves were unable to access while institutionalized, and usually voiceless.

“They did a tonne for me. Like, can you run through this book, and you want to visit the bank? Can you get the set up for when I come home? And they're doing all that stuff.” (Patient 005)

Preservation of normalcy, relations, values, and responsibilities outside of the hospital are all part of reported care partner supportive roles, and all potentially protect the identity of patients experiencing persistent critical illness.

## 4 Discussion

In this qualitative descriptive study aiming to describe care partner roles in clinical settings for adults experiencing persistent critical illness, activities supporting the physical, mental health, social, cognitive, and spiritual elements of care were reported by patients, care partners, and health care providers. Care partner roles that mitigate physical, mental health, social, and cognitive safety risks associated with persistent critical illness are discussed below, along with implications for policy, practice, and competency training.

Physical care roles such as hair washing, mobilization, and massage were among the most reported activities valued by care partners in this study. This is, perhaps, because they are the easiest to recognize and obvious to characterize as task-based actions. Supportive roles of family members at the bedside during weaning trials in the ICU have been long established and have been described by family to include acts of touch (both therapeutic and affectionate), talk (to the patient and health care team), and surveillance (interpretive and protective) (21). Similarly, a Swedish study of missed care in hospitals reported that basic care acts such as mobilization, turning, feeding, as well as health communication among the most reported care elements that were missed during inadequate staffing or urgent situations on the ward (22). Implications for missed care can include further deconditioning, skin breakdown and delays in recovery, making the work care partners do essential and physically protective for patients, particularly in high acuity clinical settings where the patient: nurse ratio is large. Thus, our results suggest the need for explicit role clarification including delineation of physical care actions that care partners are interested in and able to perform, as well as care partner training to be able to execute these roles competently and safely.

Reduction of anxiety and providing encouragement were reported as care partner roles supportive of mental health by participants in this study. Related to the emotional support provided by care partners, socially supportive roles beyond the social function of transient visitors were described. Although social isolation and living alone have been found to increase risk of hospital admission for respiratory disease, the experience of social isolation and loneliness during prolonged hospitalization and critical illness has not been well studied (23). A recent cohort study reported that social isolation before critical illness was associated with greater disability burden and higher mortality in the year following ICU admission (24). Happ et al. (21), reported persistent critical illness patients who had family present had significantly longer weaning trials than those without. The reported protective effect of the emotional and social roles of care partners during and post prolonged critical illness, though difficult to tease apart, is supported in this study with implications such as the need to reduce barriers for care partner to access and have sustainable contact with patients experiencing persistent critical illness. Furthermore, given the importance of care partner roles, a better understanding of the impact of being a care partners is needed.

Health care provider participants of this study reported that care partner roles are protective of patient cognition, and in particular, the experience delirium. A recent retrospective study of US adult ICU patients quantitatively reported a reduction in delirium duration in ICU with family presence or phone calls (25). Possibly related to the protective role of care partner on ICU delirium are the many ways in which they facilitate and support patient communication. In the cognitive domain, participants in this study also ubiquitously reported that care partners were not only the “voice” of patients when they were not awake enough to “speak” for themselves, but that they were able to interpret patient non-vocal messages and expression of needs better than anyone else because of their long-standing knowledge of the patient.

Care partners were also information brokers between patients and health care providers, which had great relevance and safety implications for patients with prolonged admissions and across multiple transitions. Family members have been known to be facilitators for patient communication during critical illness, a role sorely missed during peak visitor restrictions of the COVID-19 pandemic (26–28). Conceptualizing communication support through a safety framework for patients experiencing persistent critical illness may improve opportunities to identify and address contextual and individual level supportive interventions. Implications of these findings reinforce the need to support family presence during persistent critical illness, provide proactive and systematic communication competency training for both health care providers and care partners in these clinical settings, as well as integrate processes of care that take patient and family expertise and knowledge into account (12).

Given the breadth of the roles that care partners play in specialized clinical settings, and the multidimensional impacts on patient safety, recommendations include an urgent imperative to co-design and deploy supportive processes and policies to create sustainable care partner programs. Furthermore, an understanding of the impacts of care provision on care partners as well as implementation barriers and facilitators will strengthen the delivery of care partner programs in settings where patients with persistent critical illness are cared for.

## 5 Strengths and limitations

This study had several strengths and limitations. A strength of this study was that it included a sample of professionally diverse health care providers. It also included patients and families who were able to recall and describe their experiences with care partners during persistent critical illness. Another strength of this study was that it is the first the authors are aware of that explicitly aims to understand the roles of care partners during persistent critical illness and particularly from the perspectives of patients, care partners, and health care providers in specialized care settings.

Limitations include a self-selected sample and despite including a diverse range of participants across two hospital critical care units, the results of this single hospital study may not be generalizable to other organizations. Another limitation of this study is the self-selection bias of participants, which might limit reported experiences to include those of people with either very positive or very negative experiences. Also, as many of the participants were known to the principal investigator, we acknowledge that a limitation of this study might also include social desirability bias of participant responses.

## 6 Conclusion

In this descriptive qualitative study, we identified patient, care partners, and health care provider reported physical, mental health, cognitive, social, and spiritual roles of care partners that are protective of the safety of patients experiencing persistent critical illness. Findings from this study will contribute to the co-design, implementation, and evaluation of a formalized care partner program that is acceptable, appropriate, and feasible to implement in settings where the care of patients experiencing persistent critical illness.

## Data Availability

The original contributions presented in the study are included in the article/Supplementary Material, further inquiries can be directed to the corresponding author.
