# Supplementary material for: Reported roles of care partners in a specialized weaning centre—perspectives of patients, care partners, and health care providers
Source: Front Health Serv. 2024 Oct 30;4:1439410. doi: 10.3389/frhs.2024.1439410 (PMC11557519; doi:10.3389/frhs.2024.1439410)
Supplement: Supplementary file 1 [file Table1.docx]

**Supplementary File 1**

**Semi-structured Interview Guide**

**Health care Providers**

1. Can you describe your role at MGH and experiences working with patients and care partners (or family members) in this setting?

2. What kind of support do care partners/family members plan?

3. In what ways do HCP include care partners or family as part of the health care team?

4. What are challenges, barriers or things that make it harder to include or support care partners or family in this setting?

5. What are facilitators or things that make it easier to include or support care partners or family in this setting?

6. In what ways can the care partner program be improved?

7. In what ways did you feel the care partners or family have an impact on the patient?

8. In what ways are care partners or family different from regular visitors?

9. Is there anything else you would like to share about supporting the care partner role or families/carers in general in this setting?

**Patients and Carers**

1. Can you describe your or your loved one’s experiences as a patient care partner/carer?

2. In what ways were you/they included in the care team?

3. What were barriers or things that made it harder for you/them to be a CP?

4. What were facilitators or things that made it easier for you/them to be a CP?

5. In what ways did you feel being/having a CP benefitted your/your family member/friend’s recovery?

6. Is there anything else you would like to share about the CP role or families/carers in general in this setting?
