# Supplementary material for: Reported roles of care partners in a specialized weaning centre—perspectives of patients, care partners, and health care providers
Source: Front Health Serv. 2024 Oct 30;4:1439410. doi: 10.3389/frhs.2024.1439410 (PMC11557519; doi:10.3389/frhs.2024.1439410)
Supplement: Supplementary file 2 [file Table2.docx]

**Supplementary File 2**

**CODE BOOK**

| Domain | Definition | Code | Definition |
| --- | --- | --- | --- |
| Roles | Actions that the care partners/carer took to help patients. | Physical | Actions the care partner/carer took to support the physical care, and physical safety of the patient. Also includes ways the care/partner enhanced physical access to the items or experiences that were needed or valued by the patient. |
|  |  | Mental health | Actions the care partner/carer took to support the mental health care and experience of the patient. Includes proactive (e.g., encouragement) and reactive (e.g., emotional support) actions towards the patient. |
|  |  | Social | Action the care partner/carer took to support and manage the social needs and situation of the patient. |
|  |  | Cognitive | Actions the care partner/carer took to support cognitive needs of the patient including understanding and directing care as well as receptive and expressive components of communication. |
|  |  | Spiritual | Actions the care partner/carer took to support and manage the spiritual care of the patient, including quality of life and sense of normalcy and self in relation to others. |
