# Supplementary material for: Reported roles of care partners in a specialized weaning centre—perspectives of patients, care partners, and health care providers
Source: Front Health Serv. 2024 Oct 30;4:1439410. doi: 10.3389/frhs.2024.1439410 (PMC11557519; doi:10.3389/frhs.2024.1439410)
Supplement: Supplementary file 3 [file Table3.docx]

**Supplementary File 3**

Additional quotes from a. patient, b. carer, and c. health care provider participants.

| Code | Sub-category | Patient Quotes |
| --- | --- | --- |
| Physical | Personal care and physical therapy | “Oh, she helped me to do my therapy. Yeah. she always help me. Tell me when and how, always there.” Patient 003  “Yeah and massaged my muscles.” Patient 003  “I think no one's come to see me for 45 minutes, I need to whatever. My mom goes to get a nurse.” Patient 005  “And it, I don't know if it's it might be equal parts necessity and philosophical construct, but I'd say from the physical, from the physical point to be blunt, helping me with the bedpan or a urinal or, or you know, helping me get dressed. Getting to go out. Whatever it is. Let's be honest, I guess if it's busy, are we going to wait out an hour for a nurse, cause my mom's going to help me put my pants on.” Patient 005  “She even would wash my hair sometimes” Patient 008  “Sometimes I had a lot of pain in my feet, in my legs. And so, she would sometimes massage my feet and my calves.” Patient 008  “[write] Caregivers help with personal things like brushing teeth and hair care.” Patient 010 |
|  | Safety and surveillance | “She was so diligent about checking the meds, checking with doctors and nurses and making sure that the delivery of health care was exceptional from the hospital.” Patient 001  “I mean, she knew about my feeding tube. She knew about my ventilator. She was comfortable checking you know … my ventilator or whatever.” Patient 008 |
|  | Access to equipment and food | “They would take me outside. I love the outdoors. And they would take me outside. I could smell the flowers and feel the fresh air. It was so nice.” Patient 008 |
|  |  | “Two, the point of just being given the respect and the freedom to like to take part in the caregiver process. It's like it's, it's, it's like if I have to pee and my mom walks into the stockroom to grab a urinal, no one is going to be like "what are you doing in there". She's been there more than 3 or 4 months. So, they know her, and they trust her to do whatever she's doing out of necessity for me. So, they let her do that stuff. And it's super important because there has to be a little bit of a feeling of kind of like a make yourself at home feeling. I have to feel free to be able to grab a blanket if I'm cold or a urinal if I got to pee. And it’s the shift change so everyone is in meetings or swamped or whatever. I have to be able to feel free to do these things and my family by extension.” Patient 005  “I needed footwear, and so she got shoes for me that worked because I didn't have footwear.” Patient 008 |
|  |  | “I mean, she sometimes would bring food for me. Yeah, bring things that I like to eat.” Patient 008 |
| Mental health | Encouragement | “Well, she was here most days. She gave me support and encouragement.” Patient 001  “You should identify in my case xxx, my wife, as my greatest liaison. My, my, my greatest champion.” Patient 007  “Okay. So, I remember even she would come and watch me doing physio. And so, she would sometimes whenever I would go to the bars with [the physiotherapist], and she would just stand there and encourage me.” Patient 008  “Sure, it helped me. I can't explain to you, but when she was beside me, I felt better. Encouraging me, saying you're ok, you're not sick You're coming home. [She] made me feel hopeful.” Patient 004 |
|  | Emotional support | “My family was a real source of emotional support and strength in that with my family I could vent or discuss things that I needed to get off my chest that you might not feel comfortable with a friend or visitor. Perhaps your buddy comes over and you're not like, you know, you might not be in the mood to be like, oh man, I really hope I can get some strength back in arms. I don't want to poop in a bedpan the rest of my life. I want to be able to get on the toilet. Whereas with my mom, my sister, you're sitting there, you know, I don't want to poop in a bedpan for the rest of my life. And you know she listens when she needs to. She chimes in when she needs to. She says, let's take it slow. And they say there's a chance you're going to get substantial strength back. It's reassuring.” 005  “With everything everything she helped me. Helping my head not feel too depressed.” Patient 003 |
| Social | Companionship | “It's nice when you have somebody to talk to and it helps to have someone to talk to because you stay so long in the hospital. It was like our home. Same thing, you know what I mean.” Patient 003  “And so, if you have a parent or a sibling or spouse or someone who can advocate for you and they, you know, XXX likes this or XXX is struggling with this. Or XXX loves having this. You know, you feel like someone is advocating for you and you don't feel so alone.” Patient 008 |
|  | Gatekeeper | “She does all my dirty work telling my friends to stay away when I don't have the heart to do it.” Patient 013 |
|  |  | “My buddy was going to come over and bring some food for lunch. And he had had the flu this week. And xxx just said, you know, don't come if you're sick. She’s my guardian.” Patient 013 |
|  | Sense of community | “Yeah, but she, she knew about them [the staff] because I would talk about them. I could say so and so came and did physio with me or so and so came and gave me a bed bath and she knew who they were.” Patient 008  “I mean, she got to know the people she got to know you [the NP] and the other nurses. She chatted with them. I think that made them more, more like a family. You know, you kind of know people, and she would chat with people. And you know she would say how are you and I can say I can say, for example, oh, xxx said this, and she knew who I was talking about. So that was helpful. “Patient 008 |
| Cognitive | Health literacy, decision making, advocacy | [after CP talked to doctor about his medical condition] “Yeah, I remember that, sure. She told me that, you know she would tell me right away.” Patient 003  “Even though sometimes I might disagree. So, she'll override my decision a lot of times you're right because she knows what's better for me than me.” Patient 013  “[writes] XXX talks to the doctors and NP to get updates while I'm half-awake most of the time. He is my voice and my medical advocate.” Patient 010 |
|  | Communication support | “And I think also when you're a patient you can’t especially because while I had trouble speaking and so you can't really advocate for yourself very well. And even if you can speak every patient is different but, but you're in a vulnerable situation.” Patient 008 |
| Spiritual and quality of life | Normalcy | “Yeah, absolutely. Like I could see if I didn't get along with them. But since we get along, it's like every, like every day pretty much for the first couple of months. It's huge … with everything that happened and everything that we're enduring there's enough heaviness going on, so it's nice to keep things light. It's nice. It's nice not to have the conversation be 90% so what did the doctor say, what did the nurse say what did the physio say, are what did they say about next steps ... Let's have some what's going on at home.” Patient 005 |
|  |  |  |
|  | Support values | “So, I just see her doing her spousal, something that just comes natural to her. That I would have had my grandmother do with my grandfather. Yeah. You know, I don't know. It's just. We don't. I don't. I don't have the same emotional bond with the staff here as I do with my wife.” Patient 013  “I suffered vision loss. And I couldn't really even hold a book, so they would read to me. That was so helpful. She brought me some drawings that people, my nieces and nephews had done, and she would tape them to the wall. And I could look at them and be encouraged just by looking at them.” Patient 008 |
|  | Manage outside life | “They did a tonne for me. Like, can you run through this book, and you want to visit the bank? Can you get the set up for when I come home? And they're doing all that stuff.” Patient 005 |
|  |  | “Everything. Put it this way she's sweating right now. We're looking out the window here. Look at the possibility of rain. And she's worried about my stuff hanging on the [laundry] line outside” Patient 013 |

| Code | Sub-category | Carer Quotes |
| --- | --- | --- |
| Physical | Personal care and physical therapy | “Also, XXX developed contractions and I think had we not been there pushing him to get into his chair, communicating with him, I think he would be in worse shape than he is.” Family 006  “Because sometimes he asked me to massage his feet. And so, I do the massage for his legs, feet. And, like I said I massage his head and he fall asleep.” Family 007  “So, first I their permission, if they said yes, then I do … So, I said probably instead of waiting for the nurse to put the food, you know, the feed. Yeah, probably I can do that. Yeah. So, I said to the nurse, would you allow me to do this. Teach me how to do it. So, she teach me. So now because for him, the food is so important. It's crucial for him.” Family 007  “We did the stretch bands, and we do leg kicks. And I make sure that I get a good massage up to the knees when I do the moisturizing on the feet, stuff like that.” Family 015 |
|  | Safety and surveillance | “And number four is filling in the gaps and catching things that fall through the cracks. And that happens.” Family 002 |
|  |  | “The only thing that I said yesterday is that many, many times I saw the call bell unplugged and asked why is that?” Family 007 |
|  | Access to outside | “There are only so many man hours available to do things. Um, and so [we would do loops around [the ward].” Family 015 |
| Mental health | Encouragement | “I think that emotionally, I think I helped her just to understand sometimes what was going on and just to constantly help her to stay hopeful.” Family 009  “You know. And sometimes he said, I got tired. Okay. And I told him if you got tired, do something, move yourself. Try your best to breathe on your own so that you'll be out of that.” Family 007 |
|  | Emotional support | “And look, my daughter probably didn't want to know as much as I did, but I would use what I knew to reassure her, give her hope, help her not to be anxious.” Family 009  “So just like doing what I can to make sure her emotional well-being and happiness was there. Oh, yeah. Like bringing in, like that, uh, that little table and the coloring kit.” Family 011 |
| Social | Companionship | “Um, I mean, the primary one [role] is just keeping him company.” Family 006 |
|  | Gatekeeper | “It's also being proactive when he gets a lot of people to come and see him.” Family 012  “But the same goes with company and visitors. If someone wants to come, everybody has been very good and they will always reach out and say, can we come over? It's up to you if you don't feel like it. No one's feelings are going to be hurt. You need to be proactive. So that's where I feel like I'm stepping up and like, okay, I'll be the bulldog. You know.” Family 012  “We did ask him while he was in ICU and he didn't [want visitors], which is fine. We respected to his wishes. And then when he said he did we reached out, we asked him for the people he wanted to see first. And we've got a schedule going. And I sent information on, you know, don't come to see [him] if you have or have been exposed to a new flu or Covid and all the things that he can't handle right now. So, I'm fielding a whole lot of phone calls from people wanting to see him. I say, yes, I say he's not ready. And so, we're just we're trying to give him what he needs when he needs it and to be well informed.” Family 016 |
|  | Sense of community | “And I think the fact that. I knew team members as well. I think I could speak to people because I came to know them, and they came to know me. And I think as I felt more comfortable, it helped her to feel more comfortable. She started to feel at home.” Family 009 |
| Cognitive | Health literacy, decision making, advocacy | “Number two is I've found that I've really had to become knowledgeable about the conditions and the treatments because the case is complex and it involved multiple hospitals, multiple doctors, and particularly the continuity of care and keeping that link going has been something that I've had to do because I'm the one constant throughout the journey” Family 002  “And sometimes I would just explain a little bit of what they mean. Really. The physicians were pretty good at explaining. And [the NP was] always good at sort of interpreting what they said for us. I also did that for her sometimes, and I think that also helped her. She's also actually kind of a shy person and not one to speak up always for herself.” Family 009 |
|  | Communication support | “And I also found that when my husband couldn’t communicate either because he was on a ventilator, he had been trached before he came here. But even with the trach, he couldn't, you know, talk, but you could at least see his lips move. You know, you're really going to need that care partner to help interpret and speak for the patient, because they, in many cases, will not be able to do that for themselves.” Family 002  “So, I was kind of just teaching him and myself how to, like, communicate with each other because he didn't really use like, like anything, like any tools or any, like any writing tools. He really couldn't write either.” Family 004  “Um, some of it was just facial reactions. Quite often you're asked how does [he] express pain? And to us, if you look at his face. It's very clear when he's wincing as opposed to just sort of looking ... There's a level of I'm a little bit anxious about this concern to that really hurts, which is pretty obvious to me. But obviously I've spent 25 years looking at him. So that's probably the primary one. We sort of developed a few new hand signals, like, you know, squeeze my hand. Squeeze my hand. Yes. Things that he was pretty comfortable with and able to do so that we could continue to use yes or no questions and get answers from him.” Family 006  “Advocating for her, you know, she was not vocal most of the time. Like, I tried to do the talking for her, but even if she was, she may not be well enough, and then I kind of had to figure out what that was and communicate to the medical team.” Family 011 |
| Spiritual and quality of life | Normalcy | “So, I see myself as having 3 or 4 different roles. I mean, one of them is as a spouse and trying to be the emotional supportive partner that I would be outside of the hospital and trying to keep that as normal as possible.” Family 001  “It's just having someone in here who if nothing else is changing videos, is trying to find the way that he's comfortable in terms of what he's what's going on there” Family 006  “Yeah. Um, so I feel like that's kind of what my supportive role is and just coming and hanging out and talking and, you know, we would chat about whatever the kids or what's going on in the neighborhood or, um, he'll share something that he saw on the news or just partnership, companionship.” Family 012  “We've become very close. I love her very much. And, uh, I just try to be around and do little things every day, like foot rubs and hairdos and yeah, watching our favorite music videos and stuff like that.” Family 014 |
|  | Support values | “Other than that, there wasn't. We couldn't move anything. We couldn't touch anything. I mean, I touched him, so that's good. But I certainly we're not skilled in anything as far as all those machines are. So that was so we would, you know, I mean, in ICU, the nurses are always there. So, they heard a beep they were there. But, you know, the odd time we would go out and ask for something. And so, yeah, I guess we we just we just wanted him to feel that he's part of our family even when he couldn't communicate.” Family 016  “And number three is really taking a look at my spouse and trying to anticipate things that he needs or would make his life better and to advocate for those.” Family 002  “And it's also important to recognize that you see them the way they are now, but their care partner knows how they were before they got sick. And you know, they are you don't know whether this person is non-communicative at the best of times.” Family 002  “And so, you know, sort of trying to fill in some of this stuff about it to give you a more of a 360 view that when I say you, I'm telling the medical team that you do more of a 360 view of who this person is in the bed.” Family 002 |

| Code | Sub-code | Health care Provider Quotes |
| --- | --- | --- |
| Physical | Personal care and physical therapy | “And I don’t assign family members to do like, ambulation with my patients. If they're ambulating, then they’re pretty good. But like, just things like bed exercises.” HCP 13 |
|  |  | “They basically can take care of the ADLs like they can help feed the patients who are not able to feed themselves.” HCP 1 |
|  |  | “And also, it just helps like the, even when like changing patients, repositioning them just small ADL care. Yeah, they’re very helpful with that.” HCP 1 |
|  |  | “Usually delegate, like certain tasks to them. I mean, obviously, not any of the controlled acts, but they just take the basic idea of care if you can, you know, delegate it to them. Just to make them feel like they have, like more control over it.” HCP 1 |
|  |  | “Yeah. For example, like, if they have wound care, it’s because we have specific wound care orders, right? Because it needs to be changed, like daily every two days or as needed, right? So if we can maybe, I’m guessing, like, let them decide if it’s appropriate time to change, or like when to do certain, like, you know, certain interventions, yeah.” HCP 1 |
|  |  | “Yeah. In terms of, like, you know, scheduling things. I’m like, let’s do this at this time. Let’s do this at this time. So that way, they feel more that they’re, they’re more involved, and, and they can also assist during those times.” HCP 1 |
|  |  | “Sometimes assisting with personal care and you know, ADLs stuff.” HCP 2 |
|  |  | “[They help with] meals and whatnot.” HCP 2 |
|  |  | “Sometimes they help with turning sometimes they I’ve worked with care partners who help with you know, personal care and bathing depending on the relationship and the education around the ventilator.” HCP 4 |
|  |  | “And, and also physically for us, like they’ll help with feeding so if we teach them feeding strategies and how someone needs to be fed, they can assist with slow, careful feeding.” HCP 4 |
|  |  | “They’re really involved in their day-to-day activities, like, helping them get up helping them set up, meal times, like set up a in a chair, or if there’s, like, for one patient, mom will make sure he’s up in a chair, if we come, then he’s ready for us.” HCP 5 |
|  |  | “Yeah, seeing them as an add on like a supplement, like augmenting the care or supplementing the care.” HCP 5 |
|  |  | “So it all depends on family members, but the ones who are really involved, they do all kinds of things, including, you know, suctioning patients providing oral care, they do like, you know, passive range of motion. Sometimes they’re involved in helping with repositioning, providing, you know, ADL, or shaving, you know, their loved ones face, or you know, they’re just being there to facilitate communication, strong advocate in terms of calling if something is wrong, you know, what I mean? So it all depends.” HCP 6 |
|  |  | “Physical that is collaboration with a bedside nurse in terms of the actual physical hands on support that then that they can provide needs to be negotiated, how much do they want to provide care at the bedside, maybe assistance with feeding with, you know, changing with sitting up in a chair.” HCP 7 |
|  |  | “And the care that I see, and let me talk about the most impactful examples I’ve seen, are related to a direct physical care. Parallel care to the staff, okay.” HCP 8 |
|  |  | “Because if there are family members’, not there, then they want your help. You know. And it’s not that we don’t want to give it. We just don’t have the manpower to give it.” HCP 9 |
|  |  | “They assist with care.” HCP 10 |
|  |  | “Like they would sometimes like reposition them … I’ve seen where persons were comfortable to top the [enteral] feeds up. Just let us know that they’ve done that and stuff like that.” HCP 10 |
|  |  | “So I’m helping them learn how to turn and reposition patients, maybe do some mouth care, maybe a little bit of like a bed bath kind of tutorial as well. Yeah, and then we do some education on, like body mechanics and stuff for the families as well, just trying to make sure that they are not going to hurt themselves while they’re helping their loved ones.” HCP 11 |
|  |  | “And then there’s tasks that they could or do help with. Kind of simple like ADLs helping patients turn, reposition, mouth care. Even just like washing their face.” HCP 11 |
|  |  | “Usually it’s ambulation, if that’s what they were doing pre hospitalization and in terms of interaction with caregivers, yeah, we sometimes see the caregivers there, and then we’ll introduce them and actually we do get them involved if they feel comfortable, for example, helping us to follow up with the chair behind or doing some bed exercises with the patient when we’re not there. Or helping feed them.” HCP 13 |
|  |  | “So, what I see some caregivers do are things like assist our personal support workers with bed bath or changing diapers, feeding.” HCP 13 |
|  |  | “Oftentimes, I do use the family and give them exercises like bed exercises, teach them what to do for range of motioning for strengthening coordination activity. So, and then if they’re at the level where they actually have some functional movement and strengthen I kind of encourage the family to kind of get them to start participating in some activities like grooming like using the yonker washing your face.” HCP 14 |
|  |  | “And then in some cases, families do help, even with the physical component of improving, because if they’re at the level where they can be transferring with one person, and we train the family, they don’t have to rely on the staff. if it’s like, sometimes it’s a staffing issue where they can’t come on time, but a family is comfortable, we are able, physio and I are able to train them, then we can actually use the family member to kind of help get them in a chair, get them on the commode or whatever it is, that needs to be done.” Yeah, HCP 14 |
|  |  | “So physically, they help actually, with lifting the patient if they can, depending on you know, everybody’s mobility status and goals and risks.” HCP 4 |
|  |  | “And practically as well if they can assist, you know, nursing team, let’s say they would offer I will comb my mother’s hair today I can shave my dad or let me help with changing my them involved in their trach care trials she would feel more comfortable or I can help with a bed bath, very practical, practical tasks that they could do.” HCP 7 |
|  |  | “Yeah, so they help a little bit with the therapy. But they were like I said, I try to see if I can get, see if it fits their schedule. And it needs to be done. Like he was if its just short trials, morning or afternoon. But eventually they’ll have to do all day. And we can’t help that. But we always try to say, you know, it’s best if they’re here. Once the once the trials get longer than the hopefully the confidence level, patient will be better and won’t require as much.” HCP 009 |
|  | Safety and surveillance | “And then we can you know, like, you know, modify the care as much as possible. Right. Which has been helpful. And sometimes the family members come in, they’re more on top of things. So, things get done faster, and faster.” HCP 001 |
|  |  | “I think it can. Let me see, I think, well, a obviously if there’s someone by the bedside, and God forbid, something happens, they can, you know, reach out for help a lot faster than if somebody wasn’t there. And you know, by the time someone comes around, if a patient can’t call for help themselves, of course.” HCP 2 |
|  |  | “Um, they just, I mean, they know the patient best, right? So, if a lot of times, like we I can’t tell you, there’s been several times where I’ve had, you know, some parent or someone say, you know, there’s something wrong, there’s something wrong. And everyone’s like, no, no, no, they’re fine. They’re fine. No, no, I know them. There’s something wrong, right? And then turns out there was something wrong. So, just, you know, things like that.” HCP 002 |
|  |  | “I mean, in an ideal world, sure, you have a couple hours a day, maybe with one person, but that’s not the case, unfortunately. So these, these people have the privilege of being at bedside far longer than we do. So if we recommend something, they can either say, yes, it’s working, or it’s absolutely not working at all. And it’s helpful for them to have that experience and be present far more than we are. So I think they’re, they’re invaluable.” HCP 004 |
|  |  | “So, I think for both the patient, the team and the family member in the room, everybody feel safer when they’re there.” HCP 005 |
|  |  | “Yeah, so again, like, all depends, some of them stay overnight. Which is always appreciated by our staff, because they feel safer that you know, there’s somebody at the bedside, because these patients, they don’t communicate, right, like most of them have tracheostomy, they can’t use a call bell or having a family member, to just be there. It’s something like, you know, this feeling of like, safety is a safety feature for staff and for patient as well.” HCP 006 |
|  |  | “I think it’s important to have that information shared to our team, by care partners, and have them there as an extra set of eyes.” HCP 007 |
|  |  | “At the next level, there is a safety component and in fact, having such a persons or persons integrated in your team as an additional level of safety, you have one because early warning but also because of individual aspects of the person’s personality physicality emotions, that cannot be easily documented within the conventional charity or transfer of care compromised a transfer of shift conversations.” HCP 008 |
|  |  | “I ask them because they know the patients is a good to suction, and maybe we’ll patient down the hall to ask the family ask, is a really good to or they might tell me no, he’s like close to vomiting. They can keep an eye on that.” HCP 009 |
|  |  | “So yeah, more eyes on the patient obviously will definitely benefit safety.” HCP 009 |
|  |  | “And for the most part they are, you know, our eyes they are our essentially a part of our team because at times where this is a very hectic population and things can go sour pretty quickly. And you know, at most points they’re there to tell us this listen, this alarm went off or you know, his heart rate was this at this time, and they like work with us as a part of a team.” HCP 010 |
|  |  | “And there’s more than that, as well. So, I’ve also seen them, for example, draw attention to issues to the medical care team about changes in patient’s condition, about things from changes in respiratory rate, agitation to a skin ulcer, for example, or things like that. So, they’re also part of increasing the vigilance that has provided in the care of the patient, which I think is also critical.” HCP 012 |
|  |  | “They’re usually the first one to draw attention to any critical safety issues. Sometimes even being they have a mucus plug or they had a low desat event, or or they are not looking too good today. They’re the ones more likely to draw the first alarm signal at any sign that anything is going right.” HCP 012 |
|  |  | “You know, helping you if you look like you’re in pain, you know, getting support from the nursing staff or just making requests advocating for certain things.” HCP 004 |
|  | Access to outside | “So, the family usually I encourage them even further. If the patient’s mental health, it’s great to get out of the room, if we can set up with the oxygen needs and everything, get them to take them off the unit, if perhaps they’re stable enough, we’re just going in the hallways, I think that really, really helps kind of promote the overall well-being of the patient.” HCP 14 |
|  | Care planning and continuity of care | “Yeah, it could miss the bigger picture. And sometimes we all come together to see the bigger picture.” HCP 5 |
|  |  | “And we have whiteboards in the room that are updated daily, but they’re involved in planning, their care, scheduling, their physiotherapy, all of those things on a daily basis, we just kind of go back to the whiteboard and go over the plan of the week on Tuesdays. But other than that it’s a daily thing that they’re involved in.” HCP 3 |
|  |  | “I mean, because they, they help coordinate the patient’s care.” HCP 12 |
|  |  | “I think it depends on the individual, but sometimes they’re involved in everything, like they help coordinate when the person is going to get up.” HCP 4 |
|  |  | “And when the physical aspects of the care are related to preparation for transition to other care settings, where it’s in the ideal situation, preparation to go home and transition. Given the situation over the last decade, in which there has been different stressors on the ability to provide care moment by moment … This has filled a very needed gap.” HCP 8 |
|  |  | “They’re always included the care. So, it’s nice to have the continuity of care, you know, what they were doing there.” HCP 6 |
|  |  | “Okay so we have care partners that would want to know these things? Because they’re thinking ahead of when we go home? What does home look like if we need to do this. So, some will ask, Can you teach me how to do this? And we’ll walk them through certain things that we go through the safety guidelines, okay? How we’re doing this in terms of sterile things, there are certain things that we would love for you to do what oh, if you were not able to do it here, but if you’re going to teach, some things are okay. And stuff like that.” HCP 010 |
|  |  | “Because even like even puffers, yeah, like showing them how to do it, like when they, you know, taking a breath, or how to hold it, how many seconds stuff like that. Because most oftentimes, they go home on puffers, and for most part, people think you just go pop pop and you’re done. Right. So, it’s like, just little things, to have them included.” HCP 010 |
| Mental health | Encouragement | “They’re the encouragement and the cheerleaders in order to keep them going through everything.” HCP003 |
|  |  | “Just if you have someone there who has faith in you that, oh, you can do this, you can get over this, you can you can you know, you will get better. Just having that faith, you know, kind of, you know, keeps your hopes up.” HCP001 |
|  |  | “I think one of the things that one of the benefits for me when I go in the room, they’re just like their motivator. It’s easier to do therapy if the caregivers there, it’s easier to do some things and so when they’re around, you know, the patient will always participate of the family members not there. But they are the ones prepping them like I know one of the moms like just okay speech is coming you know, you got to give it all. This is your chance to get better. Don’t let it slip, you know. And so there’s like this person giving that extra I don’t know how to say it like extremity like pep talk.” HCP005 |
|  |  | “Also, I guess, for us, maybe a sense of another form of encouragement to the patients, when they feel like they’re down or depressed or want to give up? I would, I would resort to, I don’t know if it was a great thing, but I would resort to just using the family members as a reason to continue on.” HCP009 |
|  | Emotional support | “The other thing is support for the family member, okay, especially when we do trials, so just trach mask trials, corking trials were like if there’s a family member there. I don’t mind extending the trial a little longer. If there’s a family member there, I was asked how long are you gonna stay? We always tell our family members, you know, to help reduce the level of anxiety down for the patient. Because so I try to coordinate with a family member when they first come, when are you around when you come by that I can coordinate treatment trials with them had data for a while, but actually this one should have probably tried to do was, yeah, so because if the family member is there can, number one help encourage the patient, but number two, get them what’s it called? Distracted from, from their fear or their anxiety. So, if it’s not family members, and we try to get them distracted with media TV, computer wherever it is.” HCP009 |
|  |  | “The family members that are there help with their, with their trials, or reducing anxiety or fear or whatever.” HCP009 |
|  |  | “Well, definitely using them as an emotional support, because I think this program is, it is tough, because you’re we’re taking ICU patients that are vented and trying to progress them off event and on trach mask and hopefully decannulated is our ultimate goal. But I think through this process, like with any kind of respiratory kind of issues, there’s a huge component of anxiety involved with these patients.” HCP014 |
|  |  | “Emotional support is really important for their family members.” HCP 007 |
|  |  | “And the next one is the positive, spiritual and emotional support by the presence of a individual who, who has greater familiarity with the patient than the staff. Even though in our units, the duration of stay and care is much prolonged. Knowing a person based on past experience, based on maybe decades of friendship, partnership, living in the same abode sharing their lives is invaluable.” HCP 008 |
|  |  | “Okay, so they also as I said our population they get really get anxious to keep them calm Yeah, they help with anxiety.” HCP 010 |
|  |  | “I think that like, the probably, honestly, if they’re not going to do anything else, I appreciate them just being here to be with the patient. Because I think those patients are here for so long and not having any we see ones that don’t have anybody. And it’s particularly I think, difficult for them. So, I think that’s like a big role is them just being there for like, emotionally supportive care.” HCP 011 |
|  |  | “And I found that every care partner that I’ve worked with has been extremely supportive in the care of very complex, difficult, challenging patients who unfortunately have been in advanced stages of various diseases for a long time.” HCP 012 |
|  |  | “They’re the first ones to be next to the patient to provide emotional support. They’re going through a tough time, if they’re in distress. They’re critical in providing some kind of mental interaction and stimulation because that is something that we horribly like at our hospital, I have no idea where we don’t even have televisions anymore on the new ward. If I was a PWC patient myself, my main concern would probably be dying of boredom before I die of sepsis. So they are critical in ensuring that our patients mental health is well supported. And that’s a role that unfortunately, nobody else in the hospital can do. There’s only so many antidepressants you can give somebody. So, I think they’re critical in all of those aspects.” HCP 012 |
|  |  | “I guess, of course, the emotional support just being here.” HCP 013 |
|  |  | “Providing emotional support for the patients.” HCP 002 |
| Social | Companionship | “… and companionship as well.” HCP 002 |
|  |  | “nobody wants to be alone.” HCP 009 |
|  |  | “I think it’s very important to have people, bedside family members or friends. Because it can get really lonely in the hospital when you’re alone.” HCP 009 |
|  |  | “And emotional I think, I can’t imagine what it would be like for the people who don’t have a voice and don’t have a way of communicating to be alone at bedside. So, I can’t imagine how lovely it feels to have somebody present.” HCP 004 |
|  | Gatekeeping | “You almost feel like you’ve got a guard at the door.” HCP 002 |
|  |  | “They are also the gatekeeper for visitors, they know who comes to visit and when they know they control those people come to visit them.” HCP 005 |
| Cognitive | Health literacy, decision making, advocacy | “I would say, family members and care partners are amazing advocates, they can really, really encourage the care team and the Allied Health team, to keep the patient top of mind to continue to do these things to continue to push and drive them. And if the person has capacity to improve, that’s amazing because then we’ve, you know, had that person advocate for it, and it’s happening, maybe more than it would be otherwise, if someone seems to be, you know, maybe not as high of a priority, and they could help advocate for them to be higher, and maybe help progress them faster.” HCP 004 |
|  |  | “Downstairs in LSP. I don’t find the families as involved in the care, like hands on, but definitely directing and involved in the decision making.” HCP 005 |
|  |  | “They ask questions like we, we often referred to them if maybe the patient is a bit too sick. So, the whole conversation is directed to them. So they are the ones asking the questions for the patient.” HCP 005 |
|  |  | “Problem solving together … They’re part of the problem solving.” HCP 005 |
|  |  | “Yeah, like having them involved having them in the decision making. Yeah, like explaining to them what it is that we’re doing, and not just speaking to the patient, but we’re including whoever’s there.” HCP 010 |
|  |  | “They will, you know, advocate like say, like, say the patient, you know, like, doesn’t want to take a certain medication at this time, maybe, because it makes them you know, like, the side effects aren’t too good for them. So, they will ask us if we can, you know, like, hold it, or reschedule it, or talk to the doctor about changing it.” HCP 001 |
|  |  | “So, advocacy for sure of their loved ones.” HCP 002 |
|  |  | “Honestly, just to tell staff, you know, these patients’ routines, right, like it’s so nice to know, patients’ routines, they can tell you, you know, they don’t like to be on their left side or you know, they don’t like their arms to be positioned like that.” HCP 006 |
|  |  | “So, we are always in, in contact in terms of updating what the functional level is, what our plans might be. So just in terms of even goal setting with the family, letting the family be involved in terms of having a say of what they want to obtain, like is it like toileting it with a bedside commode chair? Like, is it ready for that are getting the family to bring in street clothes and start dressing with them? So, I think they’re very involved in this program.” HCP 14 |
|  |  | “So, I find that the just the like the soft, the connection between the nurses and ourselves, sometimes.” HCP 005 |
|  | Communication support | “So, it can be quite involved given that a high number of our ICU patients are intubated, sedated on a ventilator, so they’re not able to communicate, so identifying their substitute decision makers, which are often immediate biological family members is of high importance.” HCP 007 |
|  |  | “Assistance with communicating and receiving updates from the ICU physicians.” HCP 007 |
|  |  | “Interpretation also happens sometimes although for most medical treatment decisions, we do use interpretation services, but that can also often help for patients that don’t speak good English, or come from different cultures.” HCP 007 |
|  |  | “Definitely the trust aspect, because a lot of times they’re coming in, and they’re not even able to talk. And, and especially this is, I think, a big one being in Toronto, there’s almost always a language barrier. So, I think that that’s really important so that they can communicate properly. So, so we know what, what they need.” HCP 011 |
|  |  | “So, the families also play an important role in translating back and forth, maybe or helping the patient understand what’s going on helping us understand what’s going on with the patient.” HCP 11 |
|  |  | “Because I think there’s so many times where patients are, so they’re, they’re usually so anxious. And there’s been so many times where if the family’s not there, and we’re trying to do something, and they’re, you know, they’re really, really, like worked up about what’s happening. And then if the family can come in and explain to them, like, it completely changes the whole dynamic in the room, like every everybody calms down.” HCP 011 |
|  |  | “I mean, I would say probably one of the most critical roles that I’ve seen them do that is extremely important is to be there to absorb the information that’s being provided, changes in that medical health, changes in status, and then distill it for the patients in a way that they can also understand where they’re at, in the process of their improvement of their disease, have any complications or anything like that.” HCP 012 |
|  |  | “So, the care partners seem to be extremely crucial at bridging that communication gap as well between the caregiver and the medical team and the patient.” HCP 012 |
|  |  | “And they are also the voice of the patient. And then often patients are not able to speak for themselves, or able to navigate the system because of everything that they’re dealing with from a health-related standpoint. So they also serve in that role pretty well as well.” HCP 012 |
|  |  | “Because at the end of the day, when you talk to a patient, you propose a care plan, and it’s up to the patient to agree or disagree with or the care plan that you want to go ahead with is something that they also want to go ahead with. It’s a two-way street. And when our patients can’t properly communicate for various reasons, and they can’t be as involved in that conversation. I feel that it’s more than just informative. I think it’s also basically part of the consent for care Process on behalf of the patient that they’re providing as well. And guiding the care that’s being delivered as well. So, I don’t see as a purely informative role.” HCP 012 |
|  |  | “Basically, anything that the patient is not able to, you know, communicate by themselves actively. It’s easier for them, like for the patient to communicate with the family, and then and then the family can tell us.” HCP 001 |
|  |  | “And then you know, they know why the patient is saying no sometimes and we may not be able to easily communicate, you know.” HCP 001 |
|  |  | “We have this one patient right now who has that exact thing. He’s not able to communicate by himself. We try to read his lips. And we tried to get him to write, to write things down, but he’s not able to do much. But I noticed when the family is here, they can understand that much better. And that’s much easier.” HCP 001 |
|  |  | “And communicating with the care team. Our yeah care team, communicating with the care team about just, you know, needs wants questions. Everything basically. Yeah, let’s just also where the advocacy comes in for people who can’t necessarily speak for themselves or advocate for themselves.” HCP 002 |
|  |  | “It’s a relief I’m sure, right, to have someone who can speak for them if they can’t speak or who can advocate right. You know, for the odd one we got who doesn’t have anyone they must? I’m sure it’s very difficult.” HCP 002 |
|  |  | “They help communicate issues between the patients and their medical team extremely well, better than we can.” HCP 12 |
|  |  | “That’s a positive, I mean, I think they’re an invaluable resource in engaging with this population in particular, given that a lot of individuals don’t have a way of communicating so early on.” HCP 004 |
|  |  | “I remember once one person said I’m gonna take my time to explain to him what’s going on. The family members said that.” HCP 005 |
|  |  | “Sometimes they need to re-hear it a few times. And having the family member there is somebody another person that got the information? Yeah. And can regurgitate it, with time, having time to regurgitate.” HCP 005 |
|  | Information management | “Then again, they might know certain things about the patient’s personality, care needs. Physical medical history, things that would you know, play a role in the patient’s health and safety.” HCP 002 |
|  |  | “I’m actually thinking about the lessons learned and the certainly from my training is in the pediatric population, you listen to the parent or the partner as an independent tool, resource valued aspect of your team is picking up things that you will never pick up in the limited time you’ve had because you don’t have that years to decades of experience.” HCP 008 |
|  |  | “Recently, we had a patient whose family member, the mother, [provided us] with lots of information, background information, history. That’s one thing we have a lot of information, aside from the chart about the patient’s therapy, their course and all that stuff, too. That’s one thing is information from the family. Maybe their social history, whatever. Especially the if the if the person caregiver or the primary person is one that is always there and always been with the patient and yeah, exactly consistency.” HCP 009 |
|  |  | “It’s not just a matter of keeping them informed. But the family member at the end of the day has much more of a story about the particular patient than we do, especially once the patient has been from multiple care plans, multiple transfers. So even if we suggest the care plan, for example, from a practicality issue with the family member might say, actually, no, the patient doesn’t like X, Y, Z or W medication has been tried, and they were not agreeable to it. Or they’ve tried this in the past, or somebody has done this in the past where I don’t think that’s my family member, my care partner would agree with the plan provided because based on their beliefs, or values or something along those lines. So I think that it’s not just a matter of keeping them updated.” HCP 012 |
|  |  | “And yeah, in terms of like, other things families can do is definitely I think they’re a wealth of information because they share things of what they see that sometimes when we come in we have very limited time like we’re a snapshot of a whole day but the family could be there for like eight hours they can share us share to us Is the team like lots of information that they see what the patient’s like whether there is some delirium or where whether there is a bit of progress that we kind of missed out on. So we kind of rely on the family in terms of getting some updates, and just kind of like gauging our, our progression and our in our assessment from that.” HCP 014 |
|  |  | “It’s just understanding from a holistic perspective, in terms of how because essentially, sometimes they are their voices for us until we’re able because you do have some persons that can communicate with writing. Some can’t, yeah, right. And just knowing a bit about this person, who they were outside of, what’s happening with them right now also helps us in a way know how to care for them.” HCP 010 |
|  |  | “Because okay, even just we remember back on our other unit with a patient that he was PWC patient, and he would get upset when we would try to give him a bath. We couldn’t understand why and he couldn’t communicate with us. And then a family member told us he didn’t like having baths in the morning. Yeah, he was like a night bath kind of person. Yeah. And then even just knowing that information and moving to that point, made such a big difference. That’s not something we would have known because you’re not going to read that in a care plan. You’re not going to read that in his history, right? So having someone to come in and someone who’s there to kind of give us an understanding of who that person is makes a big of a difference.” HCP 010 |
|  |  | It does. And I was putting my position on time where I had a PWC patient, and we got along really well until I was ready to change him. And then his eyes will, you know, say something different. He wouldn’t resist, he wouldn’t stop me. But there was a language barrier as well. And when the moment that he could cork and he was able to speak to his wife, he told her ... I love my nurse and love when I have her, but I don’t, she’s a little girl. And he would he felt uncomfortable for me because he thought I was younger than I was. And he’s like, he felt uncomfortable for me doing it. He would have preferred someone older. So and I didn’t know that because I’m like, we got along so well, like I was able to do like everything. And I even I even asked us for him, like is it cultural? And she wasn’t able to like, at first she’s like, I don’t I don’t think so. Because we’re not that was just the fact that he thought I was like this little girl and it was so uncomfortable. So, when I was able to talk to him, No, this wife there and I’m like, No, it’s a part of my job when I do this, because he felt bad for this little girl coming in having to do this.” HCP 010 |
|  | Delirium management and prevention | “I think it helps with orientation … they can be involved in managing the calendar.” HCP 005 |
|  |  | “So, I guess for instance, a lot of times I would use family in terms of making sure that if there’s a cognitive piece of it to help them to reorientate to give them some cognitive stimulation activities to do with the patient just to you know, or if it’s delirium, just kind of suggestions of what to do because they’re there constantly to kind of reinforce strategies with the patient.” HCP 014 |
| Spiritual | Normalcy | “They know what they want to watch on TV, we’ve got the computers in the room, they know what they want to watch on TV, they’re there to change it when they get sick of whatever they’re watching on TV.” HCP 003 |
|  |  | “And they know what, you know, what the patient was, like, before the illness, which, which we don’t know. So, in terms of your emotional health also, so like, like, for example, if they can play their favorite, like the favorite movies, favorite TV shows, the you know, obviously, like, we have your like, we have like a limited ability to do that kind of stuff. But if family members there, they know what the patient like beforehand, they can, you know, continue doing those, and that just helps the patient stay more motivated.” HCP 001 |
|  |  | “They post their pictures of the room and change the whole atmosphere. Right? HCP 003 |
|  |  | “So, they can bring a bit of home a bit of home here. That makes the whole institution maybe feel a bit better. Like either with reporting on other people’s news, putting on the TV for them.” HCP 005 |
|  |  | “So thinking, the experience, it helps to normalize things a bit again, you know, I look at the dad that brings his son around, you know, get out of the room, go around the unit. Like, helps with maybe a bit of mental health and just kind of get away from being in an institution and really catering to like their, their leisure, recreation. You know, connect them with home, connect them with who they are.” HCP 005 |
|  |  | “So, mom is making sure that he’s following the playoffs, like his mom is making sure that he can watch the games like that he has movies that he likes that. You know. Yeah. So, he still follows what’s going on.” HCP 005 |
|  |  | “And also, to like, regarding that patient and mom in the football. I think it’s like a way for the patient to, like, know what’s going on outside of the medical setting. And even with mom when I was talking to her about it, and how we had a conversation about the playoffs.” HCP 005 |
|  |  | “Like I had an ICU family members who told me, you know, he likes his coffee, you know, in the morning, and like, some of the things that sometimes we don’t really like, we have hard time understanding. But yes, we do understand, like some of the things that family members do, including like putting coffee into PEG, but that’s the routine that I want to know that yeah. And I want our staff to know, like, you know, this family member would tell you, you know, she doesn’t like to be woken up at you know, 730, could you please let her sleep until like, nine, we want to know these things. And there’s no way we would know, unless we have somebody to advocate for them. They’re so important.” HCP 006 |
|  |  | “Like, if you’re lying in bed, you’re like, I wonder if my sister is okay. I wonder if my dad is okay, somebody to come from home and tell me you know, they’re all Okay, is my dog. Okay, like, who’s taking care of my dog?” HCP 006 |
|  |  | “Like when somebody can bring all that information to them and say, No, I just want to let you know. I went to house today. I fed your dog. Yeah, he’s fine. Just to have that kind of sense of safety as well.” HCP 006 |
|  |  | “They can also share what this person’s mannerisms are. What triggers this person? What makes this person smile, even in a sense like that? What’s the person’s fear coming in? Yeah. Right. And so that itself help us to better understand.” HCP 010 |
|  | Support values | “There is also with such engagement and designation, there is a change in the behavior of the care team. It’s not that the person has to be there to remind people to provide excellent care. But it means that there’s an intrinsic reminder of the humanity or individuality of the of the patient. And therefore, in a stressed health care environment, it reminds, it’s a reminder of the humanity of the individual and the and also humanity of the care provider in the interaction.” HCP 008 |
|  |  | “There is the spiritual aspect, we have patients that have very strong faith. And we often see family members praying with them diverse religious backgrounds, so I can think of multiple benefits.” HCP 007 |
| Other | Varies individually | “There are other ways that they can be involved. So, yeah, so I think it depends on each individual. But I would see it as more of a positive than a negative, I would say more people would probably want to be involved than not just depends to what, you know, what extent like does, what does that include? And for each person, it might be different. What their limit is.” HCP 002 |
|  |  | “Well, it all depends on family members, some family members I’ve seen, they’re here every day is like long hours. But they don’t really do much, they kind of either don’t feel comfortable. Or they find some of them find that, you know, this is not their job, it’s health care providers job. And some of them are like very much hands on, they do pretty much everything.” HCP 006 |
|  |  | “And some of them don’t stay there just like come in the morning and they go home. But it’s always good to have them at the bedside.” HCP 006 |
|  |  | “So, it really depends, we do have families that are easy to engage, that would volunteer information that are eager to work with our team, and then we have families that don’t cope well with the ICU that have a perception that, you know, we’re, we’re against them, or we’re not necessarily on their team. So it really depends. We have a wide range of feelings that these families go through emotions, you know, and scenarios, oftentimes crisis situations.” HCP 007 |
|  | Varies over time | “One of our PWC patient that we had on H7, where at one point, like the moment his wife wasn’t there, yeah, he was panicking. And it got to a point where he was saying to her, go, go, go have fun, go do what you need to do. go do you know, go to the casino. You like that go? I’m fine. It took time. But he got because then no. She built up such a rapport and trust. And so she wasn’t like she was needed, but not in the way how she used to. Right. So that team, and then we can be here but like, Okay, I’m on. I’ll do this, do this. Do this. Do this. So you go.” HCP 010 |
